# Renalase contributes to the renal protection of delayed ischaemic preconditioning *via* the regulation of hypoxia-inducible factor-1α

**DOI:** 10.1111/jcmm.12527

**Published:** 2015-03-17

**Authors:** Feng Wang, Guangyuan Zhang, Tao Xing, Zeyuan Lu, Junhui Li, Cheng Peng, Guohua Liu, Niansong Wang

**Affiliations:** a Department of Nephrology and Rheumatology, Shanghai Jiao Tong University Affiliated Sixth People's HospitalShanghai, China; b Department of Urology, Shanghai Jiao Tong University Affiliated First People's HospitalShanghai, China; c St. Vincent's HospitalMelbourne, VIC, Australia

**Keywords:** renalase, hypoxia-inducible factor, ischaemic preconditioning, ischaemia/reperfusion injury

## Abstract

Ischaemic preconditioning (IPC) attenuates acute kidney injury (AKI) from renal ischaemia reperfusion. Renalase, an amine oxidase secreted by the proximal tubule, not only degrades circulating catecholamines but also protects against renal ischaemia reperfusion injury. Here, it has been suggested that the renoprotective effect of renal IPC is partly mediated by renalase. In a model of brief intermittent renal IPC, the increased cortex renalase expression was found to last for 48 hrs. IPC significantly reduced renal tubular inflammation, necrosis and oxidative stress following renal ischaemia reperfusion injury. Such effects were attenuated by blocking renalase with an anti-renalase monoclonal antibody. We further demonstrated that renalase expression was up-regulated by hypoxia *in vitro via* an hypoxia-inducible factor (HIF)-1α mechanism. The IPC-induced up-regulation of renalase *in vivo* was also reduced by pre-treatment with an HIF-1α inhibitor, 3-(5′-Hydroxymethyl-2′-furyl)-1-benzyl indazole. In summary, the renoprotective effect of IPC is partly dependent on the renalase expression, which may be triggered by hypoxia *via* an HIF-1α mechanism. Endogenous renalase shows potential as a therapeutic agent for the prevention and treatment of AKI.

## Introduction

Renalase is a flavin adenine dinucleotide-dependent amine oxidase which has recently been suggested to be a cytokine-like protein 1. Several cells can synthesize and secrete renalase, but renal proximal tubules are the major sites where renalase originates 2,3. Renalase degrades circulatory catecholamines and regulates blood pressure, which indicates that it plays a pivotal role in the cardiovascular complications of chronic kidney disease (CKD) 4. Recent findings have shown that exogenous renalase exhibits renal protection in a mice model of renal ischaemia reperfusion (IR) injury 5,6. Whether endogenous renalase affects renal protection under the stress condition is not understood.

It is known that ischaemic preconditioning (IPC) can activate endogenous defence mechanisms that protect against a subsequent, sustained ischaemic insult 7,8. Lee and Emala reported that IPC provides both acute and delayed protection against renal IR injury in a mice model 9. The protective mechanisms of delayed IPC in the heart, brain and kidney appears to involve several mediators including protein kinase C, inducible nitric oxide synthase and hypoxia-inducible factor (HIF) 10,11. Recent data demonstrated that HIF-1α-mediated up-regulation of miR-21 was one of the involved mechanisms in the renoprotection of delayed IPC 11.

In hypoxia, HIF-1α can increase the expression of target genes through binding to the core pentanucleotide sequence (RCGTG) in the hypoxia response element (HRE) at the 5′ promoter region of the gene 12. We found that there is the pentanucleotide of HRE in the predicted promoter region of renalase gene. Therefore, it has suggested that HIF-1α could up-regulate renalase expression, which contributed to the renoprotection of delayed IPC. In this study, a rat IPC model was used to test whether renalase was induced by IPC *in vivo*, and the role of renalase in the renal protection of delayed IPC against IR injury was investigated by blocking renalase with anti-renalase antibody. Furthermore, a cell model was used to test whether renalase was regulated by HIF-1α *in vitro*.

## Materials and methods

### Rat models of delayed renal IPC and IR injury

**Figure 1:**
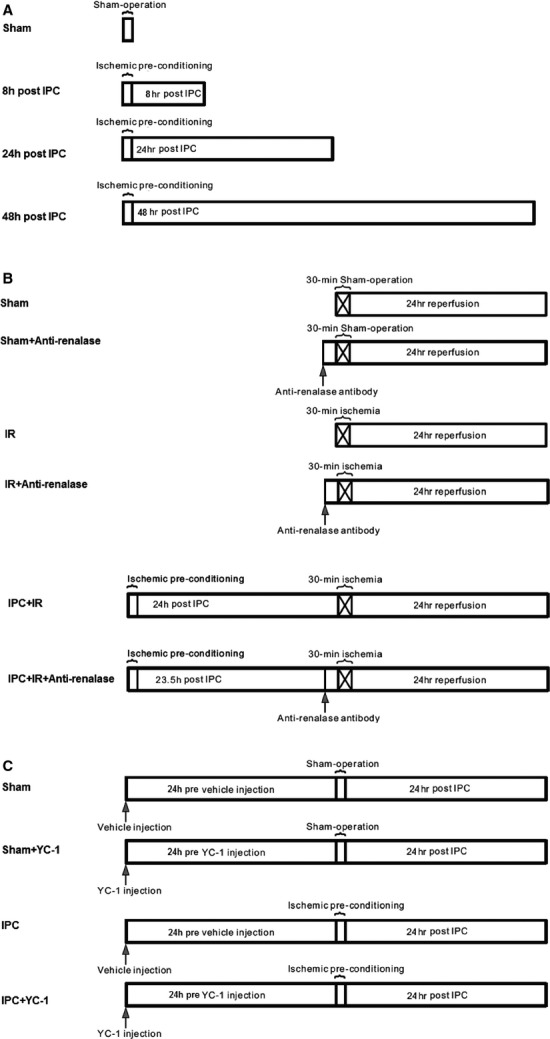
The schema for the animal experiment protocols. (A) IPC protocol. (B) IPC followed by ischaemia/reperfusion protocol. (C) IPC with HIF-1 α blocking protocol.

This study was approved by the Animal Care and Ethics Committee of Shanghai Jiao Tong University Affiliated Sixth People's Hospital. Experiments were conducted on male 6-week-old Sprague–Dawley rats (200 ± 20 g, Shanghai Science Academy Animal Center, Shanghai, China). All the animal experiment protocols were demonstrated in Figure1.

#### IPC protocol (Fig.1A)

The rats were divided into a sham-operated group (Sham) (*n* = 6) and an IPC group (*n* = 18). Rats were anaesthetized with 50 mg/kg pentobarbital. After performing a midline laparotomy, bilateral renal pedicles underwent 2-cycles of 5-min. ischaemia and 5-min. reperfusion using non-traumatic microvascular clamps. Rats were maintained at 37°C, and the abdominal cavity was hydrated with saline-moistened gauze. The rat kidneys were harvested at 8 hrs (*n* = 6), 24 hrs (*n* = 6), and 48 hrs (*n* = 6) after 2-cycles of ischaemia/reperfusion to determine the cortex levels of renalase. Rats in the Sham group underwent the same surgical procedures, except that the renal pedicles were not clamped.

#### IPC followed by ischaemia/reperfusion protocol (Fig.1B)

To investigate whether renalase contributes to the renal protection of IPC against ischaemia/reperfusion (IR) injury, the rats for IPC-IR experiment (Fig.1B) were divided into a sham-operated group (Sham) (*n* = 6), a sham-operated with anti-renalase monoclonal antibody group (Sham+anti-Ren, *n* = 6), an IR group (*n* = 6), an IR with anti-renalase antibody group (IR+anti-Ren, *n* = 6), an IR following IPC group (IPC+IR, *n* = 6) and an IR following IPC with anti-renalase antibody group (IPC+IR+anti-Ren, *n* = 6). In the IR group, rats were subjected to 30-min. occlusion of bilateral renal pedicles, followed by reperfusion for 24 hrs. Rats in anti-renalase groups were administrated with anti-renalase monoclonal antibody (2 mg/kg, dissolved in 1 ml sterilized water) at 30 min. before IR inducing or sham operation. In IPC-IR group, rats underwent IR injury after IPC and treated with the same volume of vehicle as anti-Ren groups. After IR 24 hrs, the kidneys and blood samples were collected. Rats in the Sham group underwent the same surgical procedures except the renal IR. The anti-renalase monoclonal antibody was provided by our lab, which was produced using DNA immunization and hybridoma techniques 2.

#### IPC with HIF-1 α blocking protocol (Fig.1C)

To investigate whether HIF-1α regulated renalase in IPC kidney, rats were divided into a sham-operated group (*n* = 6), an IPC group (*n* = 6), a 3-(5′-Hydroxymethyl-2′-furyl)-1-benzyl indazole (YC-1, HIF-1α inhibitor, #170632-47-0, Sigma-Aldrich, St. Louis, MO, USA) group (*n* = 6) and sham-operated animals with YC-1 group (*n* = 6). Rats in IPC group received renal IPC operation as mentioned in IPC protocol. Rats in YC-1 administrated groups received an YC-1 delivery i.p. at 2 mg/kg at 24 hrs before IPC or Sham operation, while rats in IPC group and Sham group received vehicle injection of the same volume at 24 hrs before IPC or Sham operation. Rats in the Sham group underwent the same surgical procedures, except that the renal pedicles were not clamped. The kidneys were harvested at 24 hrs after IPC.

### Renal function assessment after IR

Automatic biochemical analyser (Hitachi7600, Tokyo, Japan) was used to measure blood serum creatinine (Scr) to determine the changes of renal function.

### Histological examinations

The left kidney was fixed in 10% formalin, then dehydrated in ethanol and embedded in paraffin. Kidney tissue blocks were cut into 3-μm sections and subjected to Periodic Acid Schiff (PAS) staining. The sections were viewed by light microscopy. The histological scoring was assessed using grading tubular necrosis, loss of brush border, cast formation, and tubular dilatation in 10 randomly chosen, non-overlapping fields. The renal injury degree was estimated by the following criteria: 0, none; 1, 0–10%; 2, 11–25%; 3, 26–45%; 4, 46–75% and 5, 76–100%, as described previously 13.

A TUNEL staining for cell apoptosis was employed to assess the extent of renal apoptosis in different groups (Roche Diagnostics, Mannheim, Germany), as described previously 14.

### Lipid peroxidation of renal tissues

Malondialdehyde levels in renal tissues were determined with commercial kits following the manufacturer's protocol (Jiancheng Bioengineering Institute, Nanjing, China).

### Cell culture and hypoxia treatment

Renal proximal tubular epithelial cells from HK2 cell line (ATCC, Manassas, VA, USA) were cultured in K-SFM at 37°C 5% CO_2_, supplemented with 5 ng/ml human recombinant EGF and 0.05 μg/ml bovine pituitary extract. HK2 cells at 70–80% confluency were exposed to a hypoxia condition (2% O_2_) or 300 μM cobalt chloride (CoCl_2_, #7646-79-9; Sigma-Aldrich).

### Chromatin immunoprecipitation with anti-HIF-1α antibody from HK2 cells

Chromatin immunoprecipitation (ChIP) was performed with a ChIP kit (Millipore, Bedford, MA, USA) following the vendor's protocol. Briefly, HK2 cells were minced on ice and crosslinked with 1% formaldehyde for 10 min. after treatment with 2% O_2_ for 6 hrs. The cells were sonicated to generate chromatin fragments of 200–1000 bp that were immunoprecipitated with a HIF-1α antibody (#ab2185; Abcam, Cambridge, MA, USA) or a negative control IgG. The pullout DNA was purified using spin columns. Real-time PCR was performed to estimate the enrichment of renalase promotor DNA segments, comparing the pullout DNA (output) and input DNA samples used for immunoprecipitation. Primer sequences of predicted renalase promotor are 5′-GGTAACCTTGGGCAAACTCACTT-3′ (forward) and 5′-AGCCATAGCCCTAAAATCTCAAAAT-3′ (reverse).

### HIF decoy

Double-stranded oligodeoxynucleotides containing a hypoxia-responsive element were used as a decoy to block the activity of endogenous HIF transcriptional factor. The HIF-1α decoy sequences were 5′-GCCCTACGTGCTGTCTCA-3′ (sense) and 5′-TGAGACAGCACGTAGGGC-3′ (antisense). The scrambled oligonucleotides were 5′-GCCCTTACAACTGTCTCA-3′ (sense) and 5′-GAGACAGTTGTAAGGGC-3′ (antisense). Sense and antisense oligonucleotides were heated at 95°C for 5 min. and then cooled down slowly to room temperature 11,15. The double-stranded oligonucleotides were transfected into HK2 cells at a final concentration of 40 nM for 4 hrs using Lipofectamine 2000 (Invitrogen, Carlsbad, CA, USA). The cells were then exposed to 300 μM cobalt chloride for another 6 hrs.

### Quantitative real-time PCR

Total RNA from HK2 cells or kidney tissues was isolated using Trizol (Invitrogen). Expression levels of mRNAs were quantified in total RNA using real-time PCR with Taqman chemistry (Applied Biosystems, Carlsbad, CA, USA) as described previously 16. 18S rRNAs were used as internal normalizer for mRNAs. The primers of human renalase and rat renalase were 5′-GAAAAATCATTGCAGCCTCTCA-3′ (forward), 5′-AAGTTCTGCCTGTGCCTGTGTA-3′ (reverse), and 5′-AAAGAGGGAGATGGGTTAGTAGTGG-3′ (forward), 5′-TCGGTTCTGAGGAGGATGGAG-3′ (reverse) respectively. One-step qPCR method was used. Each reaction was performed in triplicate in clear 384-well plates at 48°C, 30 min.; 95°C, 10 min.; then 95°C, 15 sec., and 60°C, 1 min., for 40 cycles. Ct numbers (the number of cycles at which fluorescent signals reached a detection threshold that was set within the exponential phase of PCR) were used to calculate the expression levels of genes of interest normalized to endogenous cellular 18S rRNA.

### Western blot analysis

The relative protein levels of renalase and HIF-1α were analysed using Western blot analysis similar to what was described previously 17,18. The primary antibodies, anti-renalase, anti-HIF-1α, anti-β actin and anti-GAPDH were from Abcam (goat anti-renalase polyclonal antibody, ab31291, 1:500 dilution, for HK2 Western), (rabbit anti-renalase monoclonal antibody, 1:500 dilution, for rat tissues Western), Novus Biologicals (Littleton, CO, USA) (NB100-105, mouse anti-HIF-1α monoclonal antibody, 1:500 dilution), Sigma-Aldrich (A5441, mouse anti-β-actin monoclonal antibody, 1:10,000 dilution) and Santa Cruz (sc-48166, goat anti-GAPDH polyclonal antibody, 1:5000 dilution) respectively. The secondary antibodies were from Santa Cruz (horseradish peroxidase-conjugated anti-rabbit and anti-goat IgG) or Sigma-Aldrich (horseradish peroxidase-conjugated antimouse IgG). GAPDH and β-actin were used as internal control for renalase and HIF-1α respectively. All the data were obtained from ChemiDoc XRS+ System (Bio-Rad, Hercules, CA, USA) and band intensity was analysed using Image Lab 4.0.1 software.

### Statistical analysis

The statistical software SPSS (Ver. 18.0, Chicago, IL, USA) was used for data analysis. All the data were expressed as mean ± SE. One-way anova with Sidak compensation was used for parametric data and Kruskal–Wallis with Dunn' compensation for non-parametric data comparison. A value of *P* < 0.05 was considered significant.

## Results

### IPC up-regulated renalase expression

**Figure 2:**
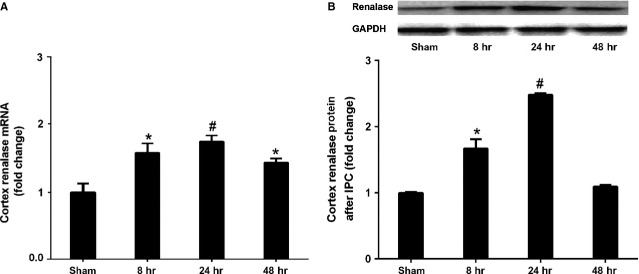
IPC up-regulated renalase expression. Rats were subjected to 2 cycles of 5 min.-renal ischaemia and 5-min. reperfusion (IPC). Kidneys were harvested at 8, 24 and 48 hrs after IPC. (A) Cortex levels of renalase mRNA; (B) cortex levels of renalase protein. *P < 0.05 *versus* control; #*P* < 0.01 *versus* control.

To examine whether IPC regulates renal renalase expression, cortical renalase levels were measured using qPCR and Western blot from IPC (2-cycles of 5-min. renal ischaemia and 5-min. reperfusion) rats. The results presented the pronounced increases of cortex renalase mRNA and protein in rats at 8, 24 and 48 hrs after renal IPC (Fig.2). These findings indicated that renal IPC could up-regulate renalase expression.

### Blocking renalase exacerbated renal IR injury following delayed IPC

**Figure 3:**
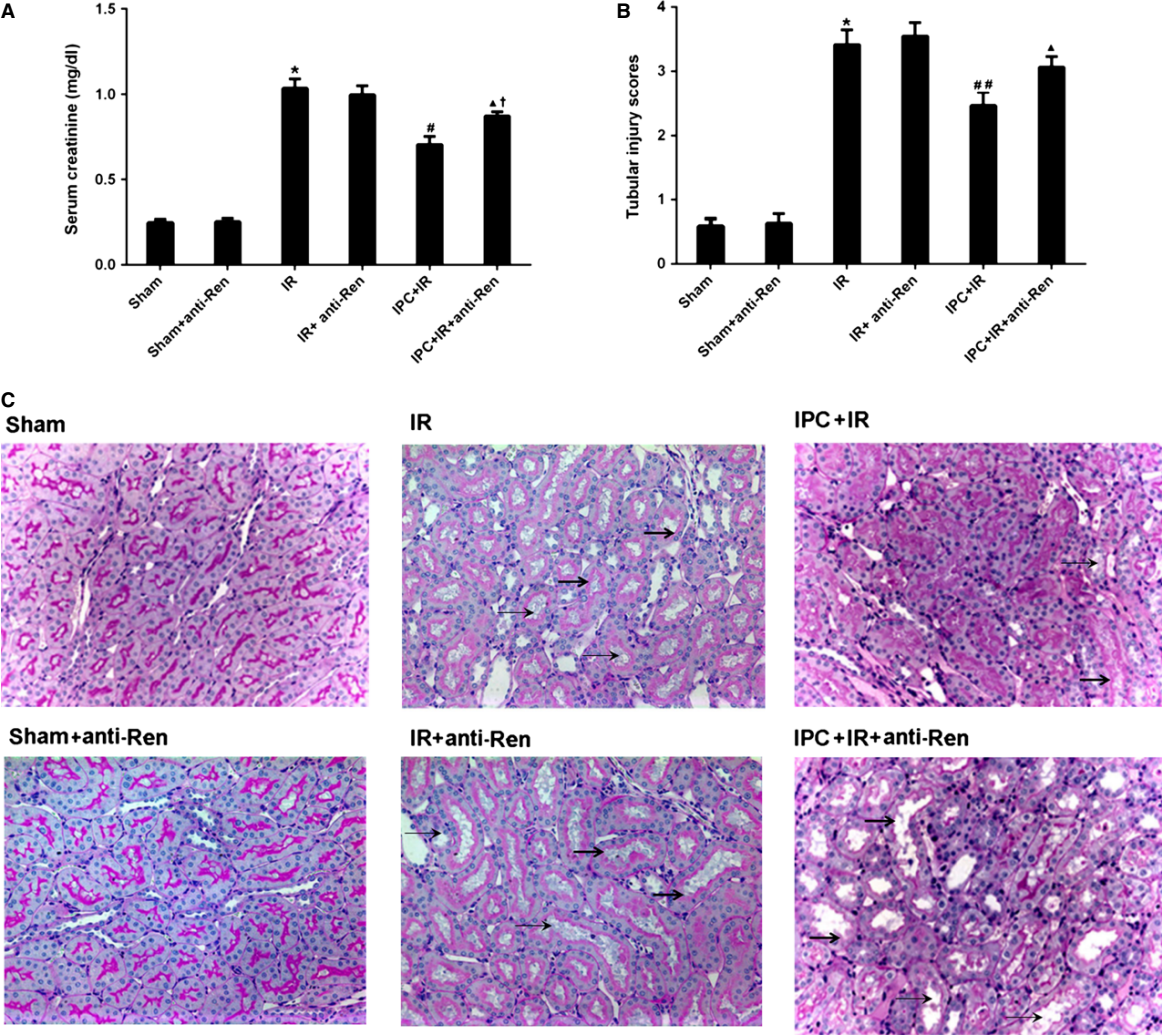
Blocking renalase exacerbated renal ischaemia/reperfusion injury following delayed IPC. Rats were subjected to 30 min. of warm ischaemia of the bilateral kidneys after IPC. Blood and tissues were collected 24 hrs after reperfusion. (A) Serum creatinine changes; (B) Tubular injury scoring; (C) Renal histological alterations (representative pictures, 200×, PAS). Sham, sham-operated control group; Sham+anti-Ren, sham-operated control group with renalase blocking; IR, renal ischaemia/reperfusion group without IPC. IR+anti-Ren, renal ischaemia/reperfusion group without IPC with renalase blocking; IPC+IR, renal ischaemia/reperfusion group following IPC. IPC+IR+anti-Ren, renal ischaemia/reperfusion group following IPC with renalase blocking. *P < 0.05 *versus* Sham; #*P* < 0.01 *versus* IR; ##*P* < 0.05 *versus* IR; ▴*P* < 0.05 *versus* IPC+IR; †*P* < 0.05 *versus* IR.

**Figure 4:**
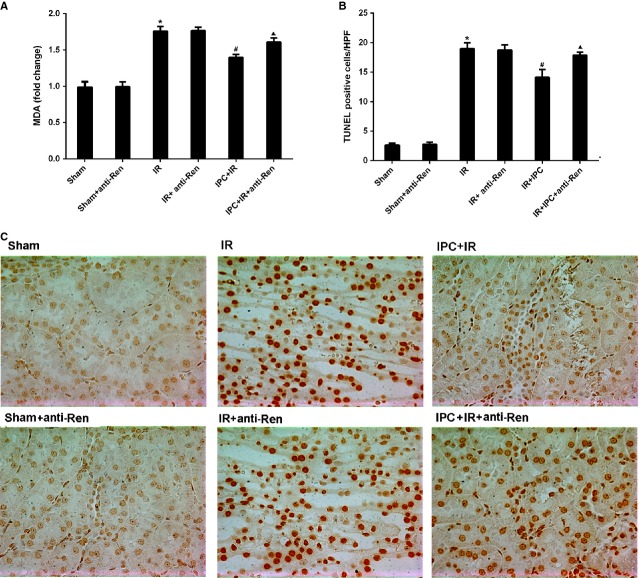
Rats were subjected to 30 min. of warm ischaemia of the bilateral kidneys after IPC. Blood and tissues were collected 24 hrs after reperfusion. (A) Cortex MDA changes; (B) Apoptotic tubular cell count; (C) Representative micrographs of TUNEL staining (400 × ). Sham, sham-operated control group; Sham+anti-Ren, sham-operated control group with renalase blocking; IR, renal ischaemia/reperfusion group without IPC. IR+anti-Ren, renal ischaemia/reperfusion group not after IPC with renalase blocking; IPC+IR, renal ischaemia/reperfusion group following IPC. IPC+IR+anti-Ren, renal ischaemia/reperfusion group following IPC with renalase blocking. *P < 0.05 *versus* Sham. #*P* < 0.01 *versus* IR; ▴*P* < 0.05 *versus* IPC+IR.

To determine whether renalase participated in the renal IPC mechanism, rat IR model after delayed IPC was established to observe the changes of the renal protection with renalase blocking. Delayed IPC exhibited significant renal protection in rats as shown in Figures3 and 4. Compared with IR control group, levels of Scr (Fig.3A), tubular scores (Fig.3B and C), cortex MDA (Fig.4A) and apoptosis (Fig.4B and C) were all reduced in IPC+IR group (*P* < 0.05 or *P* < 0.01). Levels of Scr, tubular scores, apoptosis and MDA were higher in IPC+IR+anti-Ren group than that in IPC+IR group (*P* < 0.05), which indicated that blocking renalase with a monoclonal antibody exacerbated the renal injuries (Figs3 and 4). Furthermore, there were no significant differences in renal injuries between IPC+IR+anti-ren group and IR-anti-ren group, which indicated that the renoprotection of IPC related to IPC-induced renalase strongly. However, Scr levels were still lower in IPC-IR+anti-Ren group than that in IR group (*P* < 0.05), which suggested that anti-renalase antibody abolished the protection of IPC in part. In addition, after pre-treatment with anti-renalase antibody in Sham and IR groups, no differences in levels of Scr, tubular scores, apoptosis and MDA were found. These findings indicated that renalase might at least partially contribute to the mechanism of IPC.

### Hypoxia up-regulated renalase in renal proximal tubular epithelial cells

**Figure 5:**
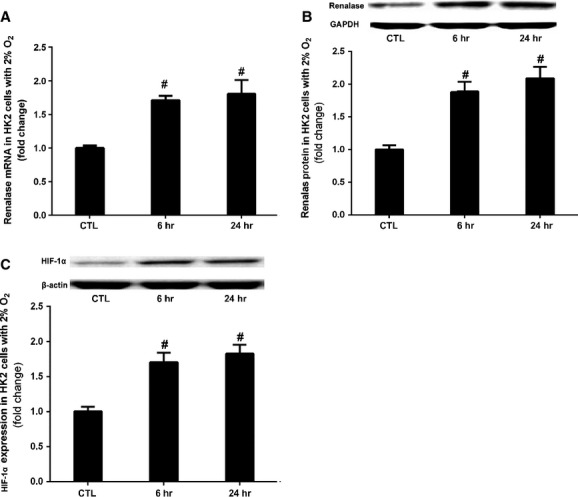
Hypoxia up-regulated renalase in renal proximal tubular epithelial cells. HK2 cells were exposed to normoxia (CTL), 2% O_2_ for 6 hrs or for 24 hrs. (A) Renalase mRNA (qPCR); (B) Renalase protein changes (Western blot); (C) HIF-1α protein (Western blot). #*P* < 0.01 *versus* CTL.

To test the hypothesis that hypoxia regulates the renalase expression, we measured the renalase expression in HK2 cells treated with low concentration oxygen. As shown in Figure5A and B both renalase mRNA and protein went up in HK2 cells exposed to 2% O_2_. Moreover, HIF-1α levels increased with renalase in HK2 cells treated with 2% O_2_ (Fig.5D). It was speculated that HIF-1α may be associated with the regulation of renalase expression.

### HIF-1α mediated renalase expression *in vitro and vivo*

**Figure 6:**
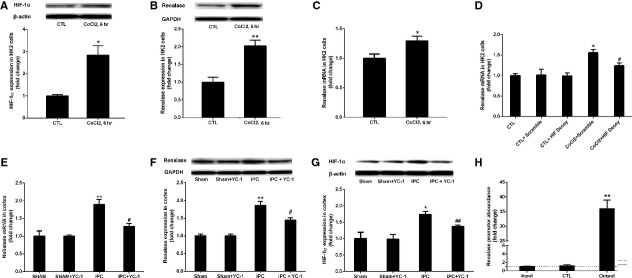
HIF-1α mediated renalase expression *in vivo and vitro*. (A) HIF-1α expression in HK2 cells treated with 300 μM CoCl_2_ for 6 hrs (Western blot); (B) Renalase protein changes in HK2 cells treated with 300 μM CoCl_2_ for 6 hrs (Western blot); (C) Renalase mRNA changes in HK2 cells treated with 300 μM CoCl_2_ for 6 hrs (qPCR). CTL, HK2 cells treated without CoCl_2_; CoCl_2_, 6 hrs, HK2 cells exposed to 300 μM CoCl_2_ for 6 hrs; *P < 0.05, **P < 0.01 *versus* CTL. (D) HIF decoy knocked down CoCl_2_-induced renalase mRNA expression *in vivo* (qPCR). CTL, HK2 cells with Lipofectamine 2000 only; CTL+Scramble, HK2 cells transfected with scramble oligonucleotides; CTL+HIF decoy, HK2 cells transfected with HIF-1α decoy; CoCl_2_+Scramble, HK2 cells exposed to CoCl_2_ after transfected with scramble oligonucleotides; CoCl_2_ + HIF decoy, HK2 cells exposed to CoCl_2_ after transfected with HIF-1α decoy. *P < 0.05 *versus* CTL+Scramble; #*P* < 0.05 *versus* CoCl_2_+Scramble. (E) Cortex renalase mRNA was down-regulated in IPC rats by YC-1(qPCR); (F) Cortex renalase protein was down-regulated in IPC rats by YC-1(Western blot); (G) Cortex HIF-1α was down-regulated in IPC rats by YC-1 (Western blot). Sham, sham-operated group; Sham+YC-1, sham-operated animal with the pre-treatment of YC-1; IPC, kidneys were harvested at 24 hrs after 2 cycles of 5-min. renal ischaemia and 5-min. reperfusion; IPC+YC-1, kidneys were harvested at 24 hrs after 2 cycles of 5-min. renal ischaemia and 5-min. reperfusion with the pre-treatment of YC-1. **P < 0.01 *versus* Sham; #*P* < 0.05 *versus* IPC; ##*P* < 0.01 *versus* IPC. (H) The enrichment of renalase promotor DNA in pullout DNA with anti-HIF-1α antibody. Input, input DNA samples; CTL, pullout DNA with a negative control IgG; Output, pullout DNA with an anti-HIF-1α antibody. **P < 0.0 *versus* CTL.

To confirm that HIF-1α regulates renalase expression, we conducted experiments *in vitro and in vivo*. *In vitro*, it was found that renalase mRNA and protein levels increased in HK2 cells after treated with 300 μM CoCl_2_ for 6 hrs, a classic HIF inducer (Fig.6B and C). Meanwhile, HIF-1α increased in HK2 cells after exposure to CoCl_2_ for 6 hrs (Fig.6A). Furthermore, with the pre-treatment of HIF decoy, renalase expression was significantly inhibited in HK2 cells comparing to the scrambled controls (*P* < 0.05) (Fig.6D). Results from *in vivo* study demonstrated highly increased renalase and HIF-1α levels in renal cortex of rats at 24 hrs after renal IPC. However, with knocking down HIF-1α with YC-1 in the delayed IPC rat model, both mRNA and protein levels of renalase were decreased as shown in Figure6E–G (*P* < 0.05).

To validate the direct regulation of renalase through HIF-1α, we carried out a ChIP approach to identify the interaction of HIF-1α with renalase genomic elements using a special anti-HIF-1α antibody. Real-time PCR amplification indicated a 36 times higher renalase DNA abundance in the pullout samples with anti-HIF-1α antibody than negative immunoglobulin controls (Fig.6H). These findings suggested that HIF-1α up-regulated renalase directly in hypoxia.

## Discussion

The present study revealed a new function of renalase in the protection against acute kidney injury (AKI) conferred by IPC. Recent findings have shown that renalase replacement may provide a novel therapeutic tool for the prevention and treatment of AKI in a mice model. Moreover, exogenous renalase attenuated renal tubular necrosis and reduced infiltrated leucocytes 5. Their studies additionally showed that renalase promoted cell survival and protected against renal IR injury in mice through the activation of intracellular signalling cascades, independent of its ability to degrade catecholamines 6. Whether endogenous renalase takes effects on renal protection under the stress condition is not understood. Our data showed that endogenous renalase plays a pivotal role in the renal protection of delayed IPC. Furthermore, another finding of this study is that HIF-1α regulates renalase expression in the kidney, which contributes to the renal protection of delayed IPC.

Acute kidney injury is a severe clinical syndrome and a major contributor to morbidity and mortality 19. IR injury is a common cause of AKI in patients undergoing acute stress such as surgery, organ transplantation, trauma, sepsis, shock, *etc*. 20. Acute IPC as well as delayed IPC provides protection against cardiac, neuronal and renal IR 9,21. However, the renal protective mechanism of IPC is not as well understood as that of cardiac and neuronal IPC 22,23. Park *et al*. reported that 15-min. prior ischaemia was partially protective against subsequent ischaemic injury 8 days later 24. Another study showed acute or delayed IPC provided renal protection against IR injury with different mechanisms 9. Our results demonstrated that IPC attenuated the renal IR 24 hrs later in rats, which is consistent with the previous reports 9. Moreover, anti-renalase antibodies partially reduced the renal protection of delayed IPC in the present study. Therefore, it is speculated that renalase might contribute to the renal protection of IPC at least in part. In addition, the renalase expression in the kidney increased profoundly induced by IPC, while the increase could be blocked by YC-1, a HIF-1a inhibitor. It can be deduced that renalase expression in cortex may be because of the regulation of HIF-1α.

Hypoxia-inducible factor-1α, a key regulator of hypoxic response, plays a crucial role in the mechanisms of IPC 25. The pentanucleotide of HRE can be found in the predicted promotor region of renalase gene. Our data *in vivo* showed that hypoxia as well as CoCl_2_ could increase the renalase expression in renal proximal tubular epithelial cells. HIF decoy can attenuate the renalase expression in HK2 cells treated with CoCl_2_. Furthermore, the ChIP results demonstrated that renalase can be bound specially by HIF-1α. In other words, findings in the present study manifested that HIF-1α might be a regulator of renalase that contributed to the renal protection of delayed IPC. On the other hand, HIF-1α is extremely liable in the absence of ischaemia/hypoxia. Thus, it would be expected that HIF levels would be restored to baseline shortly after the brief episodes of IPC. In the present study, the renalase expression peak was found at 24 hrs post-IPC. We speculated that the expression of renalase may be regulated by several transcription factors and HIF-1 may be one of them. As we described before, NF-kB pathway was also involved in its regulation 16. In addition, ischaemia could also activate the NF-kB pathway and there is a crosstalk between HIF-1 and NF-kB 26. Therefore, the sustained increase in renalase might be because of several pathways.

Renalase, a newly discovered monoamine oxidase from the kidney, can metabolize circulatory catecholamines, and the kidney is the major source of blood renalase 4,27. The discovery of renalase presents new mechanisms for the high incidence of cardiovascular complications in patients with CKD 3. Previous data exhibit that renalase decreases arterial blood pressure through oxidizing catecholamines, which is a new renal mechanism of blood pressure regulation. A renalase knockout mouse presents moderate hypertension and increased plasma catecholamines 28. According to our previous data, renalase was not secreted by podocytes or mesangial cells but by proximal tubular epithelial cells *in vitro* 2. Recent findings confer a cytokine-like property of renalase in addition to the enzymatic properties 1,5,6. This study showed that anti-renalase exacerbated the renal IR injury following IPC, which was consistent with the renal protection of exogenous renalase 5,6. Previously, we found that renalase was regulated *via* α-adrenoceptor/NF-κB pathways in renal proximal tubular epithelial cells 16. Together with the results from the present study, it can be concluded that HIF-1α is a new regulator of renalase gene. More and more data indicate that renalase is involved in not only hypertension but also heart failure, stroke, diabetes and insulin resistance 29,30. Renalase may be a valuable and effective drug to hypertension and CKD in the future 31.

Ischaemic preconditioning is becoming an effective tool to reduce IR injury because of its strong organ protection. This study indicates that renalase represents one of the mechanisms involved.
